# The interaction of online co-creative performance in a paired-player mode with creative tendency as a moderator

**DOI:** 10.3389/fpsyg.2025.1388850

**Published:** 2025-08-25

**Authors:** Ching-Lin Wu

**Affiliations:** Program of Learning Sciences, National Taiwan Normal University, Taipei, Taiwan

**Keywords:** creative tendency, divergent thinking, Remote Associates Test (RAT), Chinese Radical Remote Associates Test (CRRAT), online creativity task, moderation

## Abstract

This study investigated the moderating effect of individuals’ creative tendencies on their creative performance in interactive situations. A sample of 292 participants was selected to engage in various assessments, including the Alternative Uses Test (AUT) and the Chinese Radical Remote Associates Test (CRRAT) in single-player and paired-player modes. Additionally, participants completed the Creative Tendency Scale (CTS) as part of the data collection process. The collected performance data were used to analyze whether the creative performance of groups with different creative thinking abilities in interactive situations was moderated by creative tendency. The results revealed that risk-taking, curiosity, and total score for creative tendency moderated the relationship between CRRAT performance in single- and paired-player modes. In the context of high risk-taking, curiosity, and total score for creative tendency, the high- and low-scoring CRRAT groups showed no significant differences in their CRRAT performance in the paired-player mode. Moreover, creative tendency did not moderate the relationship between divergent thinking performance in the single- and paired-player modes. Overall, this study distinguishes the impact of creative tendency on the relationship between two types of creative problem-solving (i.e., AUT and CRRAT) in single- and paired-player modes, deepening our understanding of the connection between the cognitive and affective aspects of creativity in different contexts and how individuals exhibit their creativity in one-on-one interactive situations.

## 1 Introduction

Creativity is deemed the outcome of collective thinking and practice (Jung and Lee, 2023) and the basis for the evolution of human civilization (Thomson and Jaque, 2023). “One plus one greater than two” accurately describes the benefits of multi-person collaboration during the co-creation process (Coursey et al., 2019; Coursey et al., 2020). Team collaboration and individual work are exhibitions of creativity in interactive situations. In recent years, a few studies have focused on how individuals refine their responses in one-on-one interactive situations by referring to others (Wu, 2022) and the extrinsic factors that may influence their creativity in interactive situations (Wu and Chen, 2022). Creativity can be roughly divided into closely related cognitive and affective aspects. In other words, an individual’s ability to generate creative ideas is closely associated with creativity-related traits, such as curiosity and risk-taking (Li et al., 2022). Thus, it is reasonable to think that these traits affect creative performance in interactive situations.

Creative tendency, a typical creative trait, includes the following dimensions: risk-taking, curiosity, imagination, and a preference for complexity. These four constructs are commonly observed in highly creative individuals (Williams, 1980) and can be assessed using the Creative Tendency Scale (CTS) (Williams, 1993; Lin and Wang, 1994). Empirical studies have proven that creative tendencies are positively related to creative problem-solving (Hsiao, 2017). Therefore, creative tendencies may affect creativity in interactive situations. Examining this question would provide a deeper understanding of how the affective aspect of creativity moderates creative performance in one-on-one interactive situations, thereby advancing the theory of the co-creation process.

### 1.1 Creative problem-solving in a one-to-one interactive situation

Divergent thinking and insight problem-solving are both typical forms of creative problem solving (Wigert et al., 2024). Divergent thinking refers to the ability to form associations between remotely related ideas (fluency), produce different types of ideas (flexibility), and generate novel ideas (originality) (Torrance, 1974). Insight problem-solving occurs when an individual who first encounters impasses during the problem-solving process identifies the relationship between different parts of a problem after changing the problem’s representation and suddenly realizes the solution to their problem (that is, an “*aha!*” experience) (Weisberg, 2015; Wu et al., 2020a). These two types of creative problem-solving, which are poorly correlated (Wu, 2019), reflect the different cognitive mechanisms of creativity (Lin and Lien, 2013).

Standardized tests can assess creative problem solving (Wu et al., 1998; Huang et al., 2019; Wu et al., 2020b). The Alternative Uses Test (AUT) is a common tool that assesses divergent thinking (Torrance, 1974). In Taiwan, the commonly used standardized AUT include *Unusual Uses for Bamboo Chopsticks* (Wu et al., 1998) and *Unusual Uses for Newspapers* (Hsu et al., 2012). In addition, the Remote Associates Test (RAT) is a typical tool used to assess insight problem-solving abilities (Wu et al., 2020b). A RAT question consists of three unrelated words and requires that respondents find a word that can be paired with all three words simultaneously (Mednick, 1968; Wu and Chen, 2017).

To examine individuals’ creative problem-solving performance in interactive contexts, it is imperative to use a standardized creativity measurement tool. Online platforms are available for conducting creativity tests as developed and implemented by Wu et al. (2022). The online platform utilized in this research consisted of two creativity tests, namely, the AUT and CRRAT (Chinese Radical Remote Associates Test), each with two versions. All tests on this platform had stable internal consistency and good criterion-related validity. The platform comprises single- and paired-player modes, and can automatically compute participants’ scores. In the paired-player mode, a participant can refer to another participant’s real-time response to gather more ideas. Consequently, creative problem-solving performances in interactive situations can be observed and recorded in real time.

Current empirical studies have found that participants with lower AUT and CRRAT scores in the single-player mode achieved higher scores for fluency, flexibility, originality, and the CRRAT in the paired-player mode because they had access to another participant’s response, reducing the disparity between them and the higher scoring participants (Wu, 2022). Notably, operating contexts (i.e., cooperative or competitive) may affect creative problem-solving performance in one-on-one interactive situations. In this respect, the study indicates that participants in competitive contexts experience higher levels of competitive anxiety. Individuals perform significantly better on the CRRAT in a cooperative context than in a competitive context but show no significant differences in divergent thinking (Wu and Chen, 2022). These results indicate that other factors may moderate different types of creativity in one-on-one interactive situations. The affective aspect of creativity is closely related to creative performance (Chávez-Eakle et al., 2012), so it may play an important role in creative problem-solving performance in interactive situations. Examining this question will deepen our understanding of the factors underlying individuals’ co-creation, and the association between the cognitive and affective aspects of creativity.

### 1.2 Creative tendency and its relation to creative problem-solving

Individuals’ performance in creative problem solving reflects their cognitive creativity, while their persistent psychological traits or dispositions to creativity reflect their affective creativity (Weiss et al., 2021). Several scholars have proposed multiple traits that influence creativity. Williams (1980) summarized four common traits of highly creative people on a psychometric basis: risk-taking, curiosity, imagination, and a preference for complexity. The four traits constitute creative tendency as measured by the Creativity Assessment Packet (CAP) (Williams, 1993).

The CAP has been revised into a Chinese version (Lin and Wang, 1994), consisting of three tests: the Creative Thinking Activity, the CTS, and the Creative Thinking and Tendency Scale. The CTS evaluates creativity at the affective level, including the four traits mentioned above: risk taking, curiosity, imagination, and a preference for complexity. Risk-taking means that individuals stick to their beliefs and opinions, do not fear taking a guess or trying, and have the courage to face the unknown, criticism, or failure. As a key trait of creativity, curiosity refers to being skeptical and willing to think and ask questions to uncover the truth. Imagination refers to the ability to visualize ideas that may transcend reality and have infinite possibilities. Finally, a preference for complexity suggests that one can think logically and systematically in looking for possible solutions, even in confusing and complicated situations.

Individuals with greater creative tendencies are deemed to be more effective at utilizing their creativity, whereas those without creative tendencies are less likely to display creativity (Lin and Wang, 1994). This finding is supported by an empirical study in which Chinese elementary school children responded to divergent thinking tests and the CTS. Creative tendencies were positively correlated with divergent thinking; in particular, risk-taking and imagination (traits of creative tendency) showed higher correlations with fluency and originality (Hsiao, 2017). In addition, performance on the Chinese RAT was positively correlated with creative tendency. It had even more significant correlations with risk-taking, imagination, and a preference for complexity, reflecting a positive association between creative tendency and insight problem-solving (Jen et al., 2004). These results suggest a strong association between the cognitive and affective aspects of creativity.

Overall, creative tendency reflects willingness and intention. Empirical studies have shown that individuals with high creative tendencies perform better on divergent thinking tests (Hsiao, 2017) and Chinese RATs (Jen et al., 2004), suggesting that creative tendency may influence one’s performance in creative problem-solving.

### 1.3 The present study

Individuals’ performance in one-on-one interactive situations and influencing factors have been preliminarily explored (Wu, 2022; Wu and Chen, 2022). However, the effect of creative traits on performance remains unclear. Therefore, the present study aimed to analyze how creative tendency affects individuals’ performance on two creativity tests (the AUT and CRRAT) in both single- and paired-player modes. This study analyzed how creativity-related traits influence one’s existing creativity and their correlation with creative performance in interactive situations, with creativity test performance in the single-player mode as the predictor, performance in the paired-player mode as the dependent variable, and creative tendency as the moderating variable.

To investigate the impact of affective factors on individuals’ creative performance within one-on-one interactive contexts, performance data from the Alternative Uses Test (AUT) and the CRRAT were collected in single-player and paired-player modes. Data were collected using an online creativity test platform. In addition, the scores for each creative tendency construct were collected. The present study analyzed whether the creative performance of individuals with different creative thinking abilities in interactive situations was moderated by their creative tendency, to identify traits that influence creativity in interactive situations.

Previous studies have shown that creative tendencies are positively related to divergent thinking (Hsiao, 2017) and performance on the Chinese RAT (Jen et al., 2004). Therefore, the present study hypothesizes that creative tendencies moderate the relationship between divergent thinking (including fluency, flexibility, and originality) and CRRAT performance in single- and paired-player modes.

This study analyzes the impact of creative tendencies on creative problem-solving performance in interactive situations, hoping to clarify how individuals display creativity in interactive situations and then offer insight into the correlation between the affective and cognitive aspects of creativity in interactive situations.

## 2 Materials and methods

### 2.1 Participants

In total, 292 adults (51 males and 241 females) participated in this study. Participants were aged between 20 and 30 years, with an average age of 21.03 (*SD* = 2.72). All participants were native Chinese speakers. All participants were informed of the research and provided written informed consent before the experiment began. This study was reviewed by the Institutional Review Board (IRB) of National Taiwan Normal University.

### 2.2 Measures

Online creativity tasks and the creative tendency scale (CTS) were employed as research tools as follows:

#### 2.2.1 Online creativity task

**FIGURE 1:**
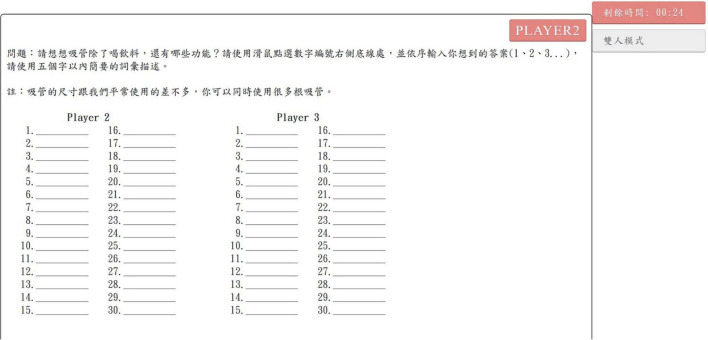
Sample of online creativity task. Note: ^┌^問題：請想想吸管除了喝飲料，還有哪些功能？請用滑鼠點選數字編號右側底線處，並依序輸入你想到的答案（1、2、3...），請使用五個字以內簡要的詞彙描述。_┘_－Question: Please think about alternative functions a straw can serve beyond drinking beverages. Click on the bottom line to the right of each number and enter your ideas sequentially (1, 2, 3...). Please keep each response concise, using no more than five words. ^┌^註：吸管的尺寸與我們平常使用的差不多，你可以同時使用很多根吸管。_┘_－Note: The straw is similar in size to a standard drinking straw. You may also consider using multiple straws simultaneously in your responses. ^┌^剩餘時間_┘_－Remaining time.^┌^雙人模式_┘_－paired-player mode.

Developed by the researcher of the present study, the real-time interactive test platform (Wu et al., 2022) contains two types of online creativity tasks: the AUT (a type of divergent thinking test) and the CRRAT (Chang et al., 2016). Its interface includes a test question section, response presentation section, response section, remaining time, and operation modes. The tasks can be undertaken in single- and paired-player modes, and one participant can refer to another ’s response in real-time in the paired-player mode. Further details are shown in Figure 1.

##### 2.2.1.1 Divergent thinking test

The divergent thinking test on this platform consisted of two versions of the AUT: *Unusual Uses for PET Bottles* and *Unusual Uses for Straws*. Respondents were scored in terms of fluency, flexibility, and originality, and their scores were computed automatically. Moreover, the test had stable inter-rater consistency (*rs* = 0.99, 0.92, 0.97, 0.97, 0.92, 0.95), good criterion-related validity (*rs* = 0.79, 0.54, 0.58, 0.75, 0.51, 0.60) with *Unusual Uses for Bamboo Chopsticks* (Wu et al., 1998) and *Unusual Uses for Newspapers* (Hsu et al., 2012), and good discriminant validity (*rs* = 0.05, 0.10, 0.14, 0.17, 0.18, 0.18) with the CRRAT.

##### 2.2.1.2 Chinese Radical Remote Associates Test (CRRAT)

The CRRAT has two forms, each comprising 20 questions based on those compiled by Chang et al. (2016). Each test question comprised three Chinese radicals, and respondents had to think of a Chinese radical that could be paired with them to create three commonly seen, legitimate Chinese characters. For instance, a CRRAT question consists of the Chinese radicals “女” (nü; female), “子” (tsu, son), and “禾” (ho; rice seedling), and one possible answer is “乃” (nai; be). Participants scored one point for each correct answer. In addition, the test had stable internal consistency (Cronbach’s α = 0.80, 0.79) and acceptable criterion-related validity with insight problem solving (*rs* = 0.48, 0.38) and CWRAT (*rs* = 0.58, 0.48) (Wu et al., 2022).

#### 2.2.2 Creative tendency scale (CTS)

The CTS in traditional Chinese used in this study was compiled by Lin and Wang (1994) based on the CAP (Williams, 1980). It consists of four dimensions assessing risk-taking, curiosity, imagination, and preference for complexity. Each dimension comprises 50 questions measured using a 3-point Likert scale. The respondents received a score for each of the four dimensions, and a total score for all four.

The validity and reliability of the Traditional Chinese CTS were examined using 2,294 samples. Its internal consistency coefficient fell between 0.40 and 0.88, test-retest reliability between 0.44 and 0.81, and inter-rater reliability between 0.88 and 0.99. The Torrance Tests of Creative Thinking were used as criterion-related validity tasks, and criterion-related validity fell between 0.26 and 0.55.

### 2.3 Procedure

Experiments were conducted in groups. The researcher explained the purpose and schedule of the experiment to the participants and asked them to sign an informed consent form prior to the experiment. Each participant took the AUT and CRRAT in single- and paired-player modes. The participants used two versions of this task. For example, respondents completed the *Unusual Uses for Straws* and CRRAT A in single-player mode, while they completed the *Unusual Uses for PET Bottles* and CRRAT B. It took them 10 min to complete the divergent thinking test and also 10 min to complete the CRRAT. After completing the above tests, the participants were asked to complete the CTS. In total, it took approximately 50 min to complete the experiment. After completing all tasks, the respondents were rewarded with NT$300 for their participation.

### 2.4 Data analysis

The researcher collated the fluency, flexibility, and creativity scores obtained in the two divergent thinking tests and their pass rates on the two forms of the CRRAT in both the single- and paired-player modes. Among the two randomly paired players, those who obtained higher scores based on their scores in the single-player mode were assigned to the high-score group. Otherwise, the participants were assigned to the low-score group. If the two players were paired to finish the tasks in the paired-player mode and had the same score, they were assigned to either group.

Subsequently, independent samples *t*-tests were used to compare the differences in AUT and CRRAT performance in the paired-player mode between the high- and low-score groups. Subsequently, we computed the product-moment correlation coefficient of creative performance for each dimension of creative tendency in the paired-player mode.

Finally, the high- and low-score groups were set as independent variables, participants’ AUT and CRRAT performance as dependent variables, and the total creative tendency score and the scores for each dimension as moderating variables. Given the relatively small sample size in this study, the bootstrapping method was employed using the PROCESS macro for SPSS (Hayes, 2018) to estimate the moderation effect by constructing confidence intervals through repeated resampling. Specifically, 292 cases were randomly resampled with replacement, generating 5,000 bootstrap samples. Based on these simulations, path coefficients and bias-corrected confidence intervals for the independent variables, moderator, and interaction term were computed. Final estimates were derived by averaging across the 5,000 iterations. When the interaction term was statistically significant, a simple slope analysis was conducted to probe the moderation effect. This involved examining the conditional effects of the independent variable on the dependent variable at values of the moderator one standard deviation above and below the mean.

## 3 Results

### 3.1 Descriptive statistics of online creative performance

Table 1 shows the means and standard deviations (SD) of the participants’ CRRAT and AUT performance (fluency, flexibility, and originality included) in the paired-player mode. First, the high-scoring CRRAT group significantly outperformed the low-scoring CRRAT group when they took the CRRAT in the paired-player mode (*t*(250) = 3.55, *p* < 0.01, *d* = 0.45). In addition, the high-scoring AUT group performed significantly better than the low-scoring group in the paired-player mode in the following three dimensions: fluency (*t*_(260)_ = 4.89, *p* < 0.01, *d* = 0.61), flexibility (*t*_(224)_ = 3.79, *p* < 0.01, *d* = 0.51), and originality (*t*(248) = 4.13, *p* < 0.01, *d* = 0.53). These results suggest that grouping based on creative performance in the single-player mode can predict differences in creative performance in the paired-player mode.

**TABLE 1:** Group difference in creativity performance.

	High-score Group			Low-score Group			*t*	*d*
	*N*	*Mean*	*SD*	*N*	*Mean*	*SD*		
CRRAT	126	0.56	0.17	126	0.48	0.22	3.55**	0.45
**Divergent thinking**								
Fluency	131	19.30	6.47	131	15.42	6.38	4.89**	0.61
Flexibility	113	9.42	2.31	113	8.23	2.39	3.79**	0.51
Originality	125	15.50	7.28	125	11.74	7.10	4.13**	0.53

**p < 0.01.

### 3.2 The relationship between creative performance and creative tendency

Table 2 lists the product-moment correlation coefficients of the participants’ performance on the CRRAT and AUT in the single- and paired-player modes to the total score of creative tendency and the respective scores for the three dimensions of creative tendency. The results indicate no significant correlation between performance on the CRRAT and the three dimensions of the creative tendency in the single-player mode (*rs* = −0.07, 0.01, −0.01, −0.06, −0.04, *ps* > 0.27). Furthermore, fluency (divergent thinking) was positively correlated with curiosity (*r* = 0.23, *p* < 0.001), imagination (*r* = 0.16, *p* = 0.008), preference for complexity (*r* = 0.14, *p* = 0.018), and the total score for creative tendency (*r* = 0.18, *p* = 0.002). Flexibility was also positively correlated to curiosity (*r* = 0.15, *p* = 0.009), preference for complexity (*r* = 0.12, *p* = 0.044), and the total score for creative tendency (*r* = 0.12, *p* = 0.041). Originality was positively correlated to curiosity (*r* = 0.19, *p* = 0.001), imagination (*r* = 0.15, *p* = 0.01), and the total score for creative tendency (*r* = 0.15, *p* = 0.011). In addition, performance on the CRRAT in the paired-player mode showed a positive correlation with curiosity (*r* = 0.15, *p* = 0.008). Fluency, flexibility, and originality of divergent thinking were significantly positively associated with all dimensions of creative tendency and total score for creative tendency (*rs* > 0.13, *ps* < 0.05). Thus, it can be concluded that there is a relatively strong correlation between creative performance in the paired-player mode and creative tendency.

**TABLE 2:** Correlations between creativity performance and creative tendency.

	RIS	CUR	IMA	PFC	CT
***Single-player mode***					
CRRAT	−0.07	0.01	−0.01	−0.06	−0.04
** Divergent thinking**					
Fluency	0.06	0.23**	0.16**	0.14*	0.18**
Flexibility	0.04	0.15**	0.08	0.12*	0.12*
Originality	0.03	0.19**	0.15**	0.10	0.15*
***Paired-player mode***					
CRRAT	0.04	0.15**	0.05	<0.01	0.08
**Divergent thinking**					
Fluency	0.19**	0.29**	0.21**	0.16**	0.25**
Flexibility	0.22**	0.22**	0.17**	0.20**	0.24**
Originality	0.14*	0.23**	0.22**	0.13*	0.22**

RIS, risk-taking; CUR, curiosity; IMA, imagination; PFC, preference for complexity; CT, creative tendency.

*p < 0.05, **p < 0.01.

### 3.3 Moderation analyses

The results for the moderating effects of creative tendency are summarized in Table 3. First of all, all three dimensions of creative tendency and its total score failed to moderate the relationship between fluency, flexibility, and originality scores in the single-player mode with those in the paired-player mode (βs = −0.08 ∼ 0.18, *ts* < 1.62, *ps* > 0.11), indicating that creative tendency had no moderating effect on the relationship between the divergent thinking performance in the single- and paired-player modes.

**TABLE 3:** Moderating effects of creative tendency on divergent thinking and CRRAT.

	Divergent thinking						CRRAT	
	Fluency		Flexibility		Originality			
	β	*t*	β	*t*	β	*t*	β	*t*
**RIS**	0.17	3.01**	0.21	3.51**	0.15	2.35*	0.02	0.38
Group	0.56	4.77**	0.50	3.86**	0.52	4.15**	0.43	3.52**
Group × RIS	−0.08	−0.68	0.16	1.35	−0.06	−0.49	−0.26	−1.99*
Sex	−0.09	−0.56	−0.06	−0.33	−0.16	−0.99	−0.15	−0.98
Age	0.00	0.03	0.02	0.63	0.00	−0.09	−0.03	0.86
R	0.34		0.33		0.30		0.28	
R^2^	0.12		0.11		0.09		0.08	
F	7.63		6.34		5.39		3.63	
**CUR**	0.26	4.70**	0.20	3.79**	0.22	3.57**	0.15	2.52*
Group	0.48	4.11**	0.45	3.49**	0.46	3.65**	0.43	3.58**
Group × CUR	0.05	0.47	0.14	1.34	−0.01	−0.11	−0.31	−2.60*
Sex	−0.11	−0.70	−0.08	−0.44	−0.19	−1.16	−0.20	−1.32
Age	0.00	0.04	0.01	0.46	0.00	−0.10	−0.02	−0.68
R	0.39		0.32		0.34		0.33	
R^2^	0.15		0.10		0.11		0.11	
F	10.19		5.62		7.94		4.89	
**IMA**	0.18	3.11**	0.14	2.18*	0.21	3.45**	0.06	0.97
Group	0.53	4.39**	0.48	3.67**	0.49	3.92**	0.43	3.51**
Group × IMA	0.08	0.67	0.15	1.14	0.02	0.16	−0.21	−1.69
Sex	−0.04	−0.27	−0.04	−0.20	−0.13	−0.80	−0.15	−0.93
Age	−0.01	−0.24	0.01	0.18	−0.01	−0.27	−0.03	−0.98
R	0.34		0.29		0.33		0.28	
R^2^	0.12		0.08		0.11		0.08	
F	7.94		4.03		7.24		3.59	
**PFC**	0.16	2.64**	0.17	2.61**	0.13	1.97*	0.03	0.56
Group	0.52	4.34**	0.47	3.56**	0.49	3.90**	0.43	3.50**
Group × PFC	0.04	0.34	0.15	1.20	−0.02	−0.17	−0.14	−1.18
Sex	−0.07	−0.46	−0.05	−0.25	−0.16	−0.95	−0.13	−0.85
Age	−0.01	−0.34	0.01	0.22	−0.01	−0.33	−0.03	−0.95
R	0.33		0.31		0.29		0.26	
R^2^	0.11		0.10		0.09		0.07	
F	6.52		5.01		4.86		3.19	
**CT**	0.23	4.11**	0.21	3.90**	0.21	3.62**	0.08	1.33
Group	0.51	4.28**	0.46	3.60**	0.48	3.84**	0.43	3.55**
Group × CT	0.02	0.22	0.18	1.62	−0.03	−0.24	−0.27	−2.22*
Sex	−0.09	−0.53	−0.05	−0.30	−0.17	−1.03	−0.16	−1.03
Age	0.00	−0.12	0.01	0.38	0.00	−0.18	−0.03	−0.82
R	0.37		0.33		0.34		0.30	
R^2^	0.14		0.11		0.11		0.09	
F	9.20		6.28		7.53		4.01	

RIS, risk-taking; CUR, curiosity; IMA, imagination; PFC, preference for complexity; CT, creative tendency.

*p < 0.05, **p < 0.01.

**FIGURE 2:**
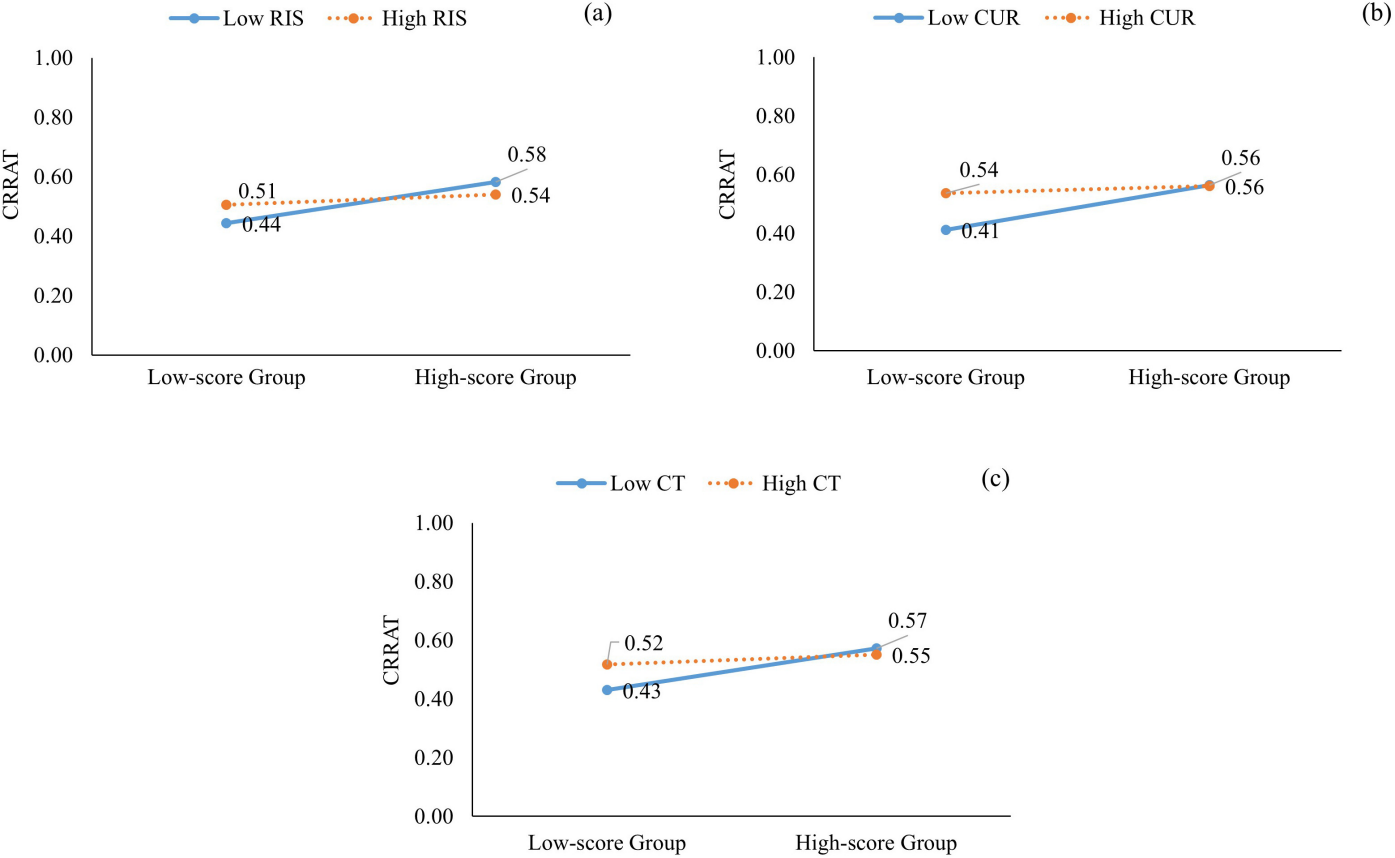
Simple slope effect of creative tendency on CRRAT. **(a)** The moderator is RIS (risk-taking); **(b)** The moderator is CUR (curiosity); **(c)** The moderator is CT (creative tendency).

Furthermore, risk-taking (β = −0.26, *t* = −1.99, *p* = 0.048), curiosity (β = −0.31, *t* = −2.60, *p* = 0.01), and the total score for creative tendency (β = −0.27, *t* = −2.22, *p* = 0.03) moderated CRRAT performance in the paired-player mode. The results of the simple slope analysis indicated that with low risk-taking (β = 0.17, *t* = 0.99, *p* = 0.33), curiosity (β = 0.12, *t* = 0.71, *p* = 0.48), and total scores for creative tendency (β = 0.16, *t* = 0.96, *p* = 0.34), individuals’ CRRAT scores in the single-player mode did not significantly predict their CRRAT performance in the paired-player mode, indicating that the high- and low-score CRRAT groups did not show significant differences in their performance in the paired-player mode. In contrast, in the context of high risk-taking (β = 0.68, *t* = 3.84, *p* < 0.01), curiosity (β = 0.74, *t* = 4.26, *p* < 0.01), and total score for creative tendency (β = 0.70, *t* = 4.03, *p* < 0.01), individuals’ CRRAT scores in the single-player mode positively predicted their performance in the paired-player mode, indicating that the high-score CRRAT group outperforms the low-score CRRAT group in the paired-player mode. In other words, those who obtained high scores in risk-taking, curiosity, and total score for creative tendency narrowed down their test score disparities in the paired-player mode, even if they had low CRRAT scores in the single-player mode. Further details are shown in Figure 2.

## 4 Discussion

This study examined whether creative tendency moderates the relationship between creative performance in single- and paired-player modes. We collected data on participants’ creative tendencies and their performance on the AUT and CRRAT in single- and paired-player modes through an online creativity test platform. The results showed that CRRAT performance in the paired-player mode was positively associated with curiosity (creative tendency), whereas fluency, flexibility, and originality (divergent thinking) were positively correlated with creative tendency and all its dimensions (risk-taking, curiosity, imagination, and preference for complexity). Furthermore, risk-taking, curiosity regarding creative tendency, and the total score for creative tendency moderated the relationship between individuals’ CRRAT performance in the single- and paired-player modes. Regarding high risk-taking, curiosity, or total for creative tendency, there was no significant difference between the performance of the high- and low-score CRRAT groups in the single- and paired-player modes.

In contrast, regarding low risk-taking, curiosity, or total score for creative tendency, the high-score group in the single-player mode still outperformed the low-score groups in the paired-player mode. Still, creative tendency did not moderate the relationship between individuals’ divergent thinking performance in the single- and paired-player modes. In other words, those who exhibited higher levels of divergent thinking in the single-player mode achieved significantly better performance in the paired-player mode, regardless of the score obtained for creative tendency and each of its dimensions. The above results reveal differing correlations between creative tendency and divergent thinking and CRRAT performance. The results also reveal the moderating effect of creative tendency on individuals’ CRRAT performance in one-to-one interactive situations.

First, the high-scoring groups significantly outperformed the low-scoring groups on the CRRAT and divergent thinking tests (fluency, flexibility, and originality) in the paired-player mode. This result suggests that in randomly paired groups, those with relatively higher levels of creativity in the single-player mode performed better in collaborative contexts regarding the quality and quantity of creative ideas they produced. This means that the collaborative mode, which allows two players to refer to each other’s responses, did not eliminate the paired participants’ disparity in creative performance, echoing the findings of previous studies (Wu, 2022). Whether other factors moderate this difference requires further investigation.

In addition, creative tendency was found to be positively correlated with divergent thinking in both modes in most cases, which partly supports previous findings with elementary school students as research participants (Hsiao, 2017). This result indicates that creativity-related traits are more positively correlated with one’s ability to associate with a wide array of unique ideas in a collaborative situation. Risk taking was significantly correlated with divergent thinking in the paired-player mode but not in the single-player mode, indicating that risk taking greatly influences creative output in the paired-player mode. Curiosity was only positively correlated with CRRAT performance in the paired-player mode. This finding is quite different from that of previous studies (Jen et al., 2004), which may be attributed to the fact that the Chinese RAT used in the cited studies was different than that used in this study. The CRRAT used in this study is akin to insight problem solving (Wu, 2019). In other words, the above results may indirectly distinguish the different correlations of creative tendency with divergent thinking and insight problem-solving.

Moreover, regarding the moderating effect of creative tendency, risk taking, curiosity, and total score for creative tendency moderated the relationship between participants’ CRRAT performance in the single- and paired-player modes. In particular, there was no significant difference in CRRAT performance between the high- and low-scoring groups in the paired-player mode when risk-taking, curiosity, or total score for creative tendency was one standard deviation above the mean. This result suggests that individuals who perform poorly on the CRRAT in the single-player mode can narrow their test score disparity in the paired-player mode if they are willing to try and face failure or think hard to find the truth (Lin and Wang, 1994). In other words, individuals brave enough to make mistakes or are willing to examine the nature of the question are more likely to improve their CRRAT performance by actively referring to others’ answers in the paired-player mode. This finding also indicates that although creative tendencies are not positively related to CRRAT performance in either single- or paired-player modes, they may play a role in moderating the relationship between CRRAT performance in both modes.

On the other hand, creative tendency did not moderate the relationship between fluency, flexibility, and originality (divergent thinking) in the single- and paired-player modes. The reason for this finding lies in the significant positive correlation between creative tendency and divergent thinking in both the single-player and paired-player modes. This suggests that regardless of an individual’s level of creative tendency, as long as they are willing to express creativity, they are capable of generating a variety of novel ideas in interactive contexts (Hsiao, 2017). Building on this observation, the present study further revealed that even individuals with high levels of creative personality traits—who can also draw on others’ responses in an interactive setting—still exhibit noticeable differences in their ability to generate diverse types of innovative ideas when paired with another participant. This indicates that individual differences in idea generation persist, despite the presence of favorable personality traits and interactive support. This finding supports the notion that the positive relationship between creative tendency and divergent thinking follows a parallel trend across individuals with varying levels of divergent thinking ability. However, it is important to emphasize that the current study examined only the moderating effect of creative tendency within one-on-one interactive scenarios. Given that creative tendency was found to be positively correlated with divergent thinking in both individual and paired contexts, it raises the question of whether creative tendency may also serve as a mediating factor in the relationship between interaction and creative performance. Future studies are encouraged to further investigate this possibility by employing mediation models to test the indirect effect of creative tendency in different interactive and individual settings.

It is worth noting that creative tendency had different effects on the relationships between insight problem solving and divergent thinking in the single- and paired-player modes, moderating the former but not the latter. This result indirectly supports the opinion that insight problem solving and divergent thinking differ in nature (Lin and Lien, 2013) and reflect different cognitive mechanisms in the paired-player mode (Wu, 2022). The CRRAT, a form of closed-ended creative problem solving with standardized answers (Chang et al., 2016), enables individuals to obtain similar test scores by referring to the answers of other participants. Furthermore, the CRRAT is moderately difficult, with a limited number of questions that can be correctly answered, which means that it is difficult to widen the test score gap between two interacting respondents. In contrast, the AUT, as an open-ended problem-solving task with no standardized answers (Wu et al., 1998; Hsu et al., 2012), allows individuals to refer to each other’s responses to come up with more creative and unusual uses in the paired-player mode, making it difficult to decrease the test score disparity between to two interacting participants regarding the quality of their responses. These results suggest that the effects of creativity-related traits may differ in a collaborative context owing to different creative problem-solving processes.

This study had three main limitations. As mentioned above, the present research set up high- and low-scoring groups by comparing respondents’ scores in the single-player mode with their randomly paired players’ scores. However, this study did not take into account other cognitive traits of participants—such as verbal intelligence and executive function—during the pairing process (Stanciu and Papasteri, 2018), nor did it include dyads in which both participants obtained identical creativity scores. One reason for this limitation is the small number of cases that met such conditions, coupled with the substantial disparity in sample sizes between high and low creative tendency groups. Future research may refine the pairing criteria to better examine how similarities or differences in creativity-related or cognitive traits influence individuals’ performance in interactive contexts. Moreover, this study assessed creativity primarily at the affective level using creative tendency as the sole indicator. Other potentially relevant characteristics or factors that could influence creative expression were not considered, such as creative personality traits, intrinsic creative motivation, and executive function. Future research could broaden the scope of assessment by incorporating additional tools such as the Creative Motivation Scale, the Creative Personality Scale, and the Delis-Kaplan Executive Function System (D-KEFS), thereby capturing a more comprehensive profile of both emotional and cognitive contributors to interactive creativity. Lastly, it is important to note that the present study focused exclusively on participants who are native speakers of traditional Chinese. As creativity can be shaped by cultural norms and values, future studies may explore how creative tendencies differ across cultural backgrounds and how such differences might affect interactive creativity. Comparative investigations involving Eastern and Western cultures, as well as individuals with monocultural versus multicultural identities, could offer valuable insights and contribute to expanding the cross-cultural understanding of creativity in social contexts.

This study is one of the first to investigate the moderating effect of creative tendency on creativity in one-on-one interactive situations. Creative tendency was positively correlated with divergent thinking but not with CRRAT performance. Furthermore, risk taking, curiosity, and total score for creative tendency moderated the CRRAT performance of both high- and low-scoring groups in one-on-one interactive contexts. In particular, under the conditions of high risk-taking, curiosity, and total score for creative tendency, the high- and low-scoring groups showed no difference in their CRRAT performance in the paired-player mode, reflecting that the variables mentioned above narrowed down the disparity between the two groups’ levels of creativity. In contrast, creative tendency did not moderate the relationship between divergent thinking in the two modes. Overall, this study distinguishes the effects of creative tendency on the relationship between the two types of creative problem-solving in both single- and paired-player modes and reveals the connection between the cognitive and affective aspects of creativity in different contexts, thus deepening the understanding of how individuals display their creativity in one-on-one interactive situations.

## Data Availability

The raw data supporting the conclusions of this article will be made available by the authors, without undue reservation.
